# The Effects of Pedestrian Environment on Ambulation with a Walking Frame in Elderly Individuals: A Survey and Experimental Study

**DOI:** 10.3390/ijerph19159327

**Published:** 2022-07-30

**Authors:** Jiemeng Yang, Chen He, Zhongjun Mo, Junchao Guo, Run Ji, Yu Wang, Chunjing Tao, Yubo Fan

**Affiliations:** 1 School of Biological Science and Medical Engineering, School of Engineering Medicine, Beijing Advanced Innovation Centre for Biomedical Engineering, Beihang University, Beijing 100191, China; yjmaline@163.com (J.Y.); jirun@nrcrta.cn (R.J.); 2 Key Laboratory of Human Motion Analysis and Rehabilitation Technology of the Ministry of Civil Affairs, Beijing Key Laboratory of Rehabilitation Technical Aids for Old-Age Disability, National Research Centre for Rehabilitation Technical Aids, Beijing 100176, China; mozhongjun@nrcrta.cn (Z.M.); guojunchao@nrcrta.cn (J.G.); 3 Laboratory of Architecture for Senior Citizen, China National Engineering Research Center for Human Settlements, China Architecture Design & Research Group, Beijing 100044, China; 6319015@cadg.cn

**Keywords:** walking aids, pedestrian environment, elderly individuals, shoulder joint, kinematics

## Abstract

Understanding the effects of sloped roads in the pedestrian environment on the body during ambulation with a walking frame can help design friendlier living environments for elderly individuals. A survey of the characteristics of walking frames used in different pedestrian environments was investigated in five communities, and a controlled study of the effects of a sloped road on a subject with different walking frames was carried out as foundational research in the laboratory. A synchronous acquisition system consisting of a wireless motion capture module and a physiological information recording module was applied to collect data on the motion of the shoulder joint and skin conductance response (SCR) of fingers in one participant. Force data were collected from sensors placed on the four legs of the walking frame. The experimental data obtained during different tasks were quantitatively analyzed. Compared to flat ground, the shoulder joint rotated in the opposite direction in horizontal and internal/external planes when using a wheeled walking frame on an uphill road, and the supportive force decreased on both uphill and downhill roads. The range of motion of the shoulder joint reduced and the direction of the shoulder joint motion changed when using a footed walking frame on both uphill and downhill roads. Additionally, the peak value of the supportive force on the uphill road appeared in the first 50% of the gait cycle, which was earlier than in the other cases. In addition, walking on the uphill road with a walking frame had a maximum SCR value, which means a greater impact of psychological arousal. Biomechanics of the shoulder joint and psychological arousal are closely related to the ease of walking on a sloped road with a walking frame. These findings are beneficial for designing more appropriate environments for elderly individuals who walk with aids.

## 1. Introduction

By 2050, the world population aged 65 and older is expected to exceed 2 billion, and the number aged 80 and older will exceed 1 billion [1]. The growth of the elderly population has become increasingly rapid all over the world [2]. Among the various special needs of elderly individuals, mobility is an important fundamental need of daily living to maintain and improve quality of life [3,4]. However, many old people suffer from motion dysfunction or limb disability with the functional degradation of the musculoskeletal system and nervous system [5].

To help elderly individuals, barrier-free environmental design has attracted increasing attention [6]. Barrier-free environments in cities and residential communities include streets, footpaths in residential areas, road nodes, crossings, public squares, etc. Road nodes include roadside stays, doorways, and entrances to communities. According to the results of a classified survey on the health status and mobility of elderly individuals, the accessibility of a neighborhood depends on its infrastructure construction and the physical and health characteristics of its inhabitants [7]. Sometimes there are precipitous situations associated with accessible facilities, such as sloped roads that affect the pathways used by elderly individuals to reach their destination, even though these are installed to help those who have any challenges in walking outside [8].

Usually, older adults with mobility difficulties choose walking aids to assist with walking [9,10]. Walking aids operated by both arms, such as walking frames or wheeled walkers that provide a stable support structure with four legs, are widely used [11,12]. When using walking aids, these devices can reduce the load on the lower limbs and improve mobility function, potentially delaying or preventing progression to mobility disability [13,14]. To compensate for the support of body weight, the load on the upper limbs is increased when using walking aids [15,16]. The joint motion angle, joint moment, and muscle activity intensity of the upper limbs also changes obviously [17,18,19]. These changes in the upper limbs can easily cause disorders, such as omalgia, osteoarthritis, and tendinitis [20,21,22].

When considering the influence of barrier-free sloped roads, the changes in human physical functional characteristics are obvious. It was found that the forward displacement of knee joints increased according to the slope of the road and that the region of muscle activation changed when walking on roads with different slopes [23]. However, few studies have examined the impact of sloped roads on the upper limb performance of older adults with disabilities or mobility impairments walking with walking aids. An experiment with a similar principle showed that the oxygen cost index, the average heart rate value, and the average driving power of wheelchair users increased significantly when propelling manual wheelchairs up a slope [24].

Another concern is that the use of walking aids may be associated with an increased fear of falling in elderly individuals [17]. Recent evidence suggests that the risk factors for elderly individuals include ascending and descending a slope, walking with or without aids, difficulty walking, and so on [25]. The fear of falling leads to elderly individuals being unwilling to walk in their home or in an outside environment [22,26]. The skin conductance response (SCR), as the most established approach, can help to understand psychological arousal [27]. Electrodermal activity means that there is variation in the electrical properties of the skin in response to sweat secretion [28]. Sweat glands are innervated by the sympathetic nerves, and sympathetic nerves are accompanied by psychological processes, including emotional arousal [29]. Increased activity of the sympathetic nervous system activates the eccrine sweat gland and increases skin conductance [29,30]. Typically, skin conductance is measured from the surfaces of the fingers or the palms of the hand [30].

Taking all these factors into account, it could be hypothesized that the sloped roads in pedestrian environments have effects on the psychology and biomechanics of the shoulder joint during walking with a walking frame in elderly individuals. To investigate this, a survey on the relationship between the pedestrian behavior of elderly individuals and community environments was conducted in this study. Additionally, a laboratory experiment was carried out to quantify the effects of sloped roads on the psychology and physiology of elderly individuals with a walking frame. The two kinds of walking aids that use both arms, a wheeled walking frame (WW) and a footed walking frame (FW), were compared with independent walking (IW) under different pedestrian conditions, including flat ground, uphill, and downhill. The force on the walking frame and joint motion of the shoulder were measured to assess the biomechanical features and SCR was measured to assess the psychological features in elderly individuals.

## 2. Materials and Methods

### 2.1. Investigation of Pedestrian Environment Encountered by Elderly Individuals Walking with a Walking Frame

A survey of the pedestrian environments of elderly individuals walking with aids was conducted in five communities. The information on the surveyed communities is listed in Table 1. Through observations, conversations, and summary records regarding the actual pedestrian network, as described in Table 2, the various problems that disturbed elderly individuals were identified and analyzed.

**Figure 1:**
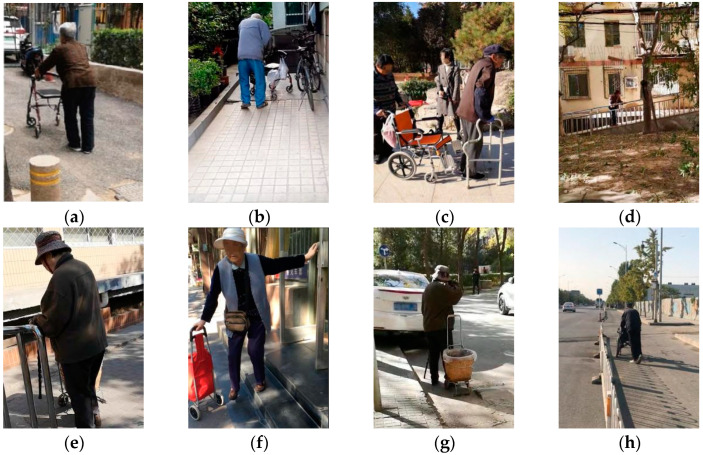
Physical activities of elderly individuals using walking aids. Including: (**a**) on level ground, (**b**) on uneven ground, (**c**) getting up after sitting down for a long time, (**d**) going uphill and downhill, (**e**) climbing the stairs, (**f**) carrying heavy objects, (**g**) obstacles, and (**h**) walking for a long time.

The walking characteristics of 220 elderly individuals with walking aids in pedestrian environments were recorded, as shown in Figure 1. Additionally, characteristics of elderly individuals with walking aids in different pedestrian environments, such as on level ground, on uneven ground, going uphill or downhill, climbing the stairs, obstacles, carrying heavy objects, getting up after sitting down for a long time, and a long duration of walking, were summarized.

### 2.2. Quantitative Research in a Typical Pedestrian Environment

Based on the investigation of and research on pedestrian environments encountered by elderly individuals, the sloped road was chosen as a typical environment due to changes in the spatial position, kinematics, and biomechanics of the upper and lower limbs, muscle activity, and psychological arousal of elderly individuals during ambulation with a walking frame.

#### 2.2.1. Experimental Materials

**Figure 2:**
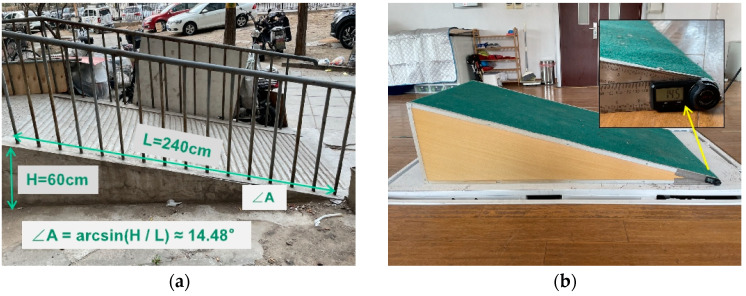
The degree of slope in the barrier-free environment: (**a**) measured in an old community, and (**b**) simulated in the experimental environment.

Pedestrian environments where walking aids can be used were assessed, and it was found that the slope of ramps was approximately 10° in newer communities or parks. However, in some old communities, the slope of the barrier-free ramp was relatively steep. The test platform was built in the laboratory to reproduce an outdoor barrier-free sloped road. Based on the maximum slope of 14.48° measured in the community, the gradient of the test platform was designed to be 14.50° with a length of 1.55 m and a height of 0.39 m for this experiment, as shown in Figure 2a,b.

Considering the influence of sloped roads on walking in elderly individuals, a healthy participant was recruited in the initial experiment to imitate elderly individuals walking with aids. The participant, with a height of 1.76 m, weight of 70 kg, and age of 24 years, signed an informed consent form before the experiment and had no head trauma, nerve, muscle, bone or joint disease, or relevant medical history that impacted daily activities.

**Figure 3:**
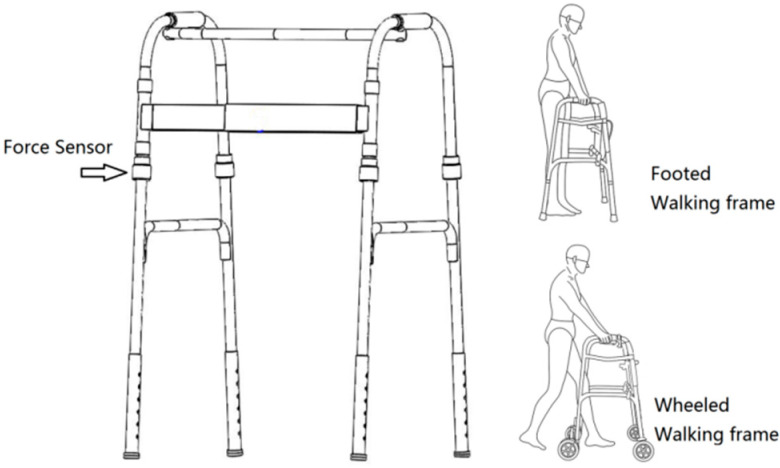
Two types of walking frames used for the experiment.

As shown in Figure 3, a standard walking frame (YC8202, Fushide, Zhongshan, China) was employed in this experiment. Its four feet could be replaced by four wheels to make a wheeled walking frame, which helped eliminate error in the experiment due to the use of walking aids with different structures. Four three-dimensional force sensors were placed on its four legs.

#### 2.2.2. Experimental Methods

The participant was trained to walk with two types of walking frames before the formal experiment. The height of the walking frame was set at the same height as the ulnar styloid during standing [31]. When using an FW, the user lifts the frame off the ground and places it in a suitable position in front of himself and then moves forward [13]. While using a WW, the user needs to push the frame forward at a comfortable gait speed [11,17]. The subject was required to adapt to using different walking aids on flat roads, uphill slopes, and downhill slopes. After the subject was familiar with the different conditions, the experiment officially began. The nine experimental tasks were carried out, including three categories: (1) walking independently on a flat road, uphill slope, and downhill slope, (2) walking with an FW on a flat road, uphill slope, and downhill slope, and (3) walking with a WW on a flat road, uphill slope, and downhill slope. The participant was allowed to have an adequate rest between each task. Each task was repeated at least 6 times to ensure that 6 groups of valid data were recorded.

**Figure 4:**
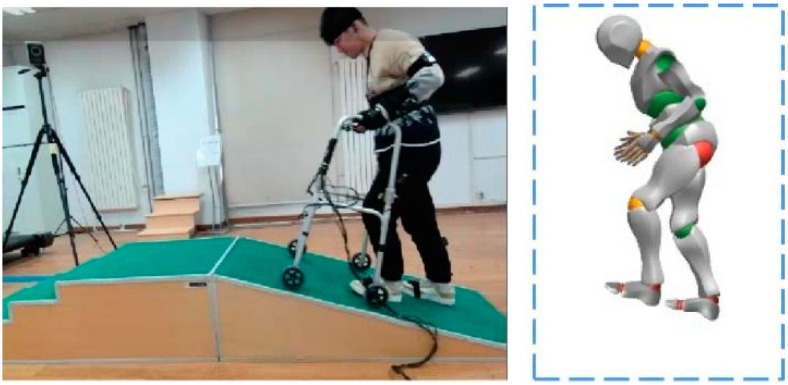
Data acquisition methods for the different experimental tasks.

A wireless motion and physiological acquisition system (CAPTIV-L7000 Premier software, TEA Ergo Inc., Vandœuvre-lès-Nancy, France) was applied in the experiment. It is a comprehensive system that continuously records a series of motion and physiological parameters of the human body in real time. Fifteen motion sensors were fixed on the subject’s head, back, waist, left and right upper arms, left and right lower arms, left and right thighs, left and right lower legs, left and right hands, and left and right feet, according to the Hanavan model [32]. SCR is typically measured using electrodes placed on the fingertips or hands, as these areas reflect increased sympathetic activity, and SCR can be assessed in response to a variety of stimuli [33]. In this study, two electrodes were attached to the index and middle fingers of the left hand. The motion sampling frequency was 32 Hz, and the SCR sampling frequency was 64 Hz. The data were processed and analyzed with the ErgoLAB 3.0 software (TEA Ergo Inc., Vandœuvre-lès-Nancy, France). As shown in Figure 4, the participant completed the task, and the model simulated human motion in real time via this software. The force sensors measured the forces of the three axes with a sampling frequency of 100 Hz. Considering that the maximum supportive force appeared in the vertical direction [34], the z-axis force was studied first in this study. In this experiment, the gait cycle began with the left heel touching the ground and ended with the left heel touching the ground again. The relationship curves were obtained by normalizing and calculating the mean of the 6 valid experimental datasets for each task with MATLAB R2018b software (The MathWorks, Inc., Natick, MA, United States).

## 3. Results

### 3.1. Empirical Results from the Community

#### 3.1.1. Problems with the Environment for Elderly Individuals

As listed in Table 3, the barrier-free environment problems that obviously affected elderly individuals who walked with a walking frame according to the surveys and interviews were summarized as four points.

#### 3.1.2. Behaviors of Elderly Individuals in the Different Cases

The results of the survey show that the usefulness of walking aids to elderly individuals was mainly affected by the environmental conditions and individual changes in state when starting to move. The elderly people displayed the behaviors described in Table 4 in seven common-use cases. Accordingly, it could be found that sloped roads are one of the more special pedestrian environments that cause some elderly individuals to face mobility problems, and they may even need assistance from others.

### 3.2. A Controlled Study Results in a Laboratory Scenario

Based on the above findings, this experiment selected a representative spatial scenario (doorway) and a representative use of elderly individuals using walking aids (going uphill and downhill) to be examined quantitatively in the laboratory. In this experiment, the motion characteristics of the shoulder joint, the load on the walking aids, and the SCR of the body were analyzed to learn more about the usefulness of walking aids when elderly people are moving in pedestrian environments.

#### 3.2.1. Shoulder Motion Characteristics

**Figure 5:**
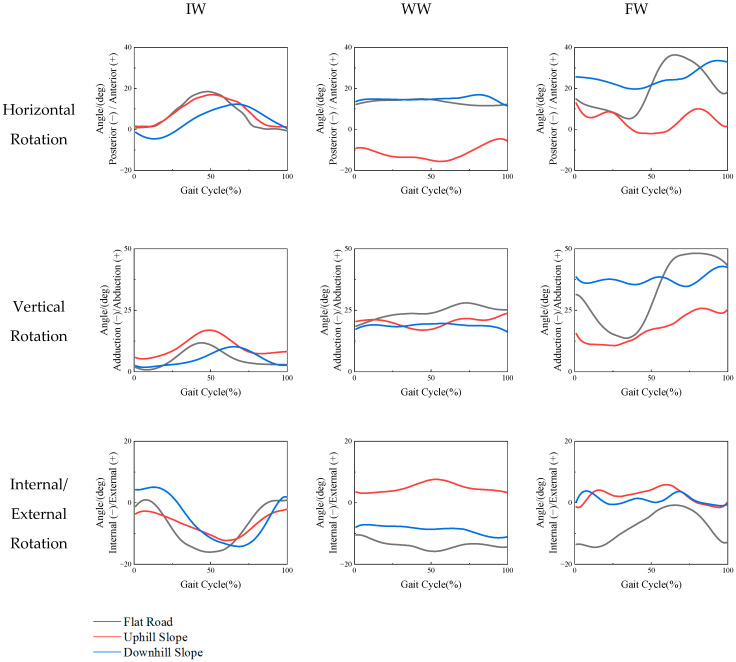
Comparison of the effects of different tasks on the motion of the shoulder joint.

Based on the results obtained, joint angle−gait cycle relationship curves were generated for the horizontal rotation, vertical rotation, and internal/external rotation, as shown in Figure 5. The comparison of angles was based on the shoulder motion of a subject walking on the flat road. Overall, compared with unassisted walking, spatial changes made more obvious the influence on shoulder joint motion when participants walked with aids.

It was logical that the shoulder motion of the man going uphill with a WW was more affected in the horizontal rotation plane with a peak posterior angle of 15.60° and in the internal/external rotation plane with a peak external angle of 7.63°. With FW ambulation on the uphill and downhill slopes, the kinematic patterns of the shoulder in the horizontal and vertical planes were obviously changed compared with those observed on the flat road. Additionally, the motion angles of the shoulder joint when walking downhill were greater than those when walking uphill.

#### 3.2.2. Supporting Force Characteristics

**Figure 6:**
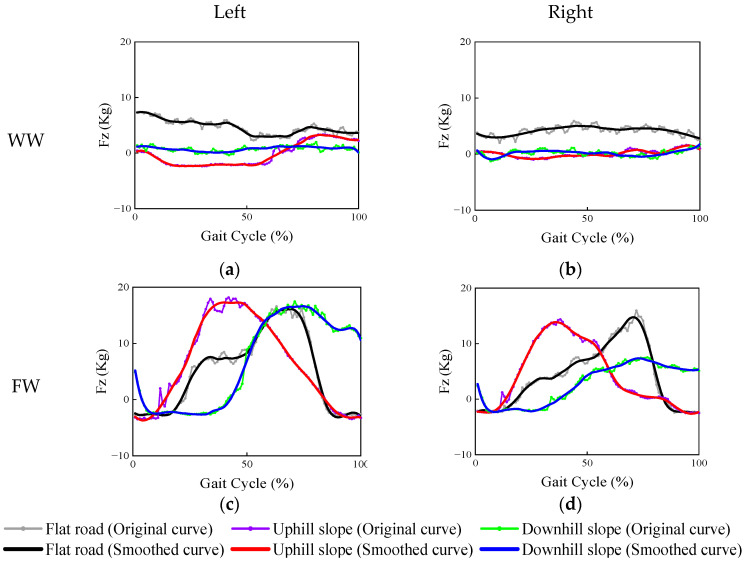
Supporting force on both sides of the walking aids, including: (**a**) force on the left side of the WW; (**b**) force on the right side of the WW; (**c**) force on the left side of the FW; and (**d**) force on the right side of the FW.

The load value–gait cycle curves in the z-axis direction for the two types of walking frames are illustrated in Figure 6. These curves represent the reaction forces exerted on the upper extremities in the direction of support. There were significant differences between the two types of walking frames, evident in force values that changed sharply when an FW was employed. The WW could support up to 15.97% of body weight (∑Fz = 11.18 kg), and the FW could support up to 44.17% of body weight (∑Fz = 30.92 kg) when walking on a flat road. According to Figure 6, the WW provided less supportive force on both sides when used to aid uphill or downhill walking. In contrast, the FW provided a greater supportive force on both sides. From Figure 6c,d, the peak load with the FW on the uphill road appeared in the first half of the gait cycle, which was earlier than it appeared during walking on the flat or downhill road.

#### 3.2.3. SCR Characteristics

**Figure 7:**
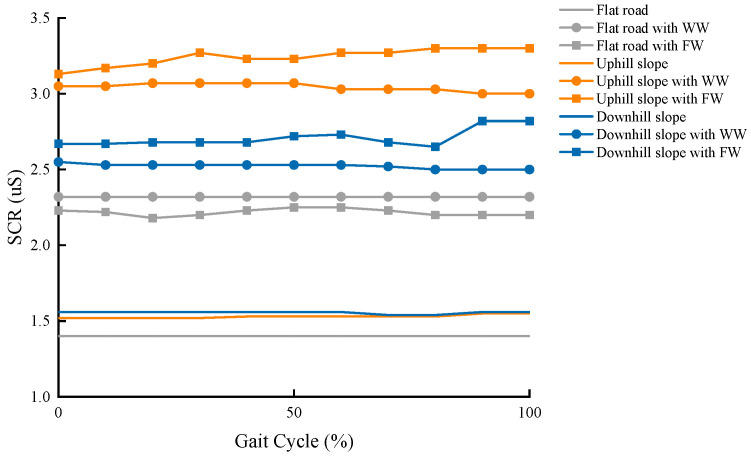
Comparison of the SCR observed during different tasks.

The changes in SCR after the human body received the command to start walking are shown in Figure 7 for one gait cycle. The task stimulation indicated that there were noticeable differences in SC between the participants walking with and without walking aids. The factors affecting SC from most to least were uphill, downhill, and flat road slopes. It also suggested that the use of an FW caused SC to increase more than a WW when the user walked on a slope, and the average difference was approximately 0.19 μS for the uphill slope and 0.17 μS for the downhill slope.

## 4. Discussion

Building a barrier-free pedestrian environment for an aging population that uses walking aids is important for fostering independence and health [35]. The main purpose of this study was to analyze how different sloped pedestrian environments influenced elderly individuals when they needed a walking frame to assist them.

From the investigation of the living environment of elderly individuals, many problems were found that affect the interaction between the individuals and their environment, specifically their barrier-free environment. The environmental conditions and the changes in state of elderly individuals affected the usefulness of walking aids. The more surprising finding was that sloped walkways, a barrier-free design element in the community, actually make it difficult for elderly people to use a walking frame to assist them during walking. Therefore, this situation was reproduced in the laboratory for further quantitative analysis.

The pedestrian environment in the experimental study was divided into flat, uphill, and downhill roads, and the slope angle adopted was the maximum value measured in the living environment of elderly individuals. A WW and FW were employed separately in the tasks. From the perspective of human functional compensation, the burden on the upper limbs was increased because the purpose of using a walking frame is to reduce the burden on the lower limbs, which is likely to cause shoulder pain [36,37]. At the same time, some elderly people may have special feelings when using walking aids, such as a fear of falling [22,38]. Therefore, this study investigated the biomechanics of the shoulder joint and psychological arousal characteristics of the walking-assisted locomotion methods.

The experimental tasks were first conducted on the flat ground with the FW and WW. The ranges of shoulder joint motion were found to compare favorably with results reported in earlier studies. When the WW was employed, the range of motion in the shoulder joint was decreased in three planes, which was consistent with the characteristics of shoulder joint motion described by Guo [17]. Compared with the normal range (180°) of the shoulder joint abduction [39], there was 9.44° and about 5.24% of the normal range of shoulder abduction when walking with a WW. Additionally, as it was approximately similar to the study of Konop [40], being about 2.59% of the normal range of shoulder abduction also demonstrated the characteristic that the range of shoulder joint motion was obviously decreased with WW. When the FW was employed, the shoulder joint angle exhibited obvious changes in the horizontal and vertical planes with the distance between the subject and the FW. The shoulder was abducted and internally rotated, and the maximum angle of shoulder abduction occurred at 75% of the gait cycle. These characteristics of the relationship curves of the shoulder joint motion angle–gait cycle were consistent with previous studies [41,42,43]. With respect to the supporting force in the vertical direction, Pardo [44] reported that the peak vertical forces of a standard walking frame ranged from 33 to 67% body weight. In the present study, the peak of the supporting force was about 44.17% body weight, which fit the point of Pardo. Based on these, the results could be considered to assess the effects of sloped roads on shoulder joint biomechanics when using a walking frame.

The results of the shoulder joint motion analysis indicate that sloped roads have effects on people who use walking aids. When the participant walked without a frame, the direction and angle of the shoulder joint motion during uphill and downhill walking were approximately the same as those on flat ground, and only the peak value of the joint angle when walking downhill appeared later than in the other two cases. In contrast, the effects on the shoulder were different when a WW or FW was used.

On one hand, the influence of the WW on the shoulder joint is mainly found in the range and the direction of motion. When the subject walked with a WW on the downhill slope, the motion pattern was similar to that on flat ground, and only the internal rotation angle decreased slightly. However, when the subject walked with the WW on the uphill road, the shoulder turned posteriorly and externally, which is contrary to the shoulder motion during the flat and downhill conditions. This might be due to the shoulder not only playing a supporting role as a connecting joint when using a WW, but also overcoming the weight of the WW to move forward on the uphill road. Therefore, the motion patterns are obviously different.

On the other hand, under the three road conditions, the angle and direction of shoulder joint motion are clearly different when an FW was employed. Both uphill and downhill slopes affected shoulder motion when the FW was employed. The shoulder joint almost maintains anterior rotation in the horizontal plane and abduction in the vertical plane, which leads to a decrease in the range of shoulder rotation. It can also be found that the increase in the joint angle on downhill slopes is greater than that on uphill slopes. Based on the comparison of the joint motion data, it can be proven that the barrier-free slope changed the pattern of shoulder motion in elderly individuals who needed an FW. In addition, elderly individuals need to lift their FW and move it forward during walking, which leads to balance problems, especially on slopes. Therefore, they have to control the angle of the upper limb motion to increase their support area on uneven ground.

The current study provides a comparison of the loads in the vertical direction on both sides of the walking frame as a reacting force of the support provided. When the WW was used, the supportive force on the flat road was greater than that on the sloped road. It is considered that the force in the vertical direction can be divided into two parts when pushing the WW on the uphill and downhill slope. One part is used to support the weight of the body, and the other part is used to control the WW. Meanwhile, it is interesting to note that when the FW was operated, the peak supportive force appeared earlier on the uphill slope, and the force attenuation was slower on the downhill slope. It is supposed that the dependence on the FW is considerable on the uphill slope because the body needs to overcome the weight of both itself and the FW. However, on the downhill slope, the actuation duration is prolonged as a result of controlling the speed and maintaining balance.

In line with previous studies [13,22,25,26], the use of walking aids can lead to psychological changes. However, classified studies based on different barrier-free roads are limited. Based on the technique of skin conductance analysis to characterize psychological arousal, this work also discussed changes in psychology when using walking frames during different tasks. After giving the same “walk” order, the skin electrical data of the participant were recorded for one gait cycle. It is important to note that an uphill slope made the psychological arousal of the person more obvious, whether the person was walking with the WW or FW. It is considered that uphill walking requires more power from the human body, thus the psychological arousal would be more evident. From this standpoint, the presence of an uphill slope can be regarded as an important factor affecting elderly people who use walking aids.

There still are some limitations in this study. First, the degradation of physical function is personalized in the elderly and it is difficult to be controlled in the laboratory, which interferes with the experimental results. To minimize the influence of individual factors on experimental results and clearly study the effects of the pedestrian environment on joint motion, a young, healthy subject participated in the tasks. Second, the installation of the four force sensors added weight to the walking frame, which led to experimental errors. To decrease the weight difference, the attachments of the walking frame were removed, such as the seat on the walking frame. Third, only the change of the supportive force in the z-axis direction was analyzed and compared in the initial results, and the variation force in the other two directions on the walking frame will be described next. In the future, the classified research according to the physiological characteristics of the elderly will be carried out. Meanwhile, the analysis of the shoulder joint stress characteristics based on motion data and force values will be conducted to discover optimal methods for the design of walking frames.

## 5. Conclusions

This study was conducted to investigate the effects of sloped roads in pedestrian environments on the psychology and biomechanics of the shoulder joint during walking with a walking frame in elderly individuals. Based on the study results, whether elderly individuals walk with a WW or an FW on sloped roads, the shoulder motion characteristics, reaction force required to support the body weight, and even the psychological arousal of the individual are obviously different from those observed when walking on flat roads. These findings are beneficial for future designs of appropriate pedestrian environments for elderly individuals with walking aids.

## Figures and Tables

**Table 1:** The information of five surveyed communities.

Number	Year	Area (Hectares)	Number of Buildings	Number of Public Activity Spaces
Community 1	1997	24	23 multi-story residential buildings and 5 high-rise residential buildings	6
Community 2	1991	19	18 multi-story residential buildings and 8 high-rise residential buildings	4
Community 3	1990	8	9 multi-story residential buildings and 3 high-rise residential buildings	2
Community 4	1995	14	21 multi-story residential buildings and no high-rise residential buildings	4
Community 5	1988	17	17 multi-story residential buildings and 8 high-rise residential buildings	2

**Table 2:** The results of a survey form of community pedestrian environments and problems.

Pedestrian Environments	Problems
Roads in the community	Coexistence of people and vehicles
	Sidewalks are occupied
	Narrow sidewalk
	Deceleration belt hinders walking
	Drainage grate is close to deceleration belt
Entrance of buildings	No sloped walkway at the building entrance
	Inappropriate gradient of sloped road
	Sloped roads are occupied
	Aisle spaces are occupied
	Sloped road is directly connected to vehicle road
	Barrier-free sloped road with pipe shaft
Public activity Spaces	A height difference between the space and the sidewalk
	Sloped roads are occupied by vehicles
	Uneven ground
	Paving of small facing bricks on the ground
	Dazzling lights on the ground
	Uneven pavement caused by water pipes

**Table 3:** The problems of barrier-free environments for elderly individuals during walking.

Environment Problems	Performance
Incoherent and disorganized accessibility	Accessible designs that directed elderly individuals to their destination are often not coherent enough. Generally, the following need to be properly connected: entrance to the residential building → roads → entrance to the destination or public square.
Poor traffic layouts	Mixed modality routes were used by pedestrians and vehicles; some sidewalks are too narrow; and more than half of a sidewalk was blocked by trees. As a result, there might be insufficient space for an elderly person to use a walking aid.
Not enough attention is given to the details and there may be hidden dangers	The key parts of some accessible routes were poorly designed and may contain hidden dangers.
Poor or disorganized management of public spaces	Ramps were frequently blocked, which hinders the travel of elderly individuals.

**Table 4:** Characteristics of the elderly with walking aids in different pedestrian environments.

Cases	Behaviors of Elderly Individuals
On level ground	Some elderly people can remain upright when using a walker, whereas others, due to their poorer sense of balance, lean their upper body toward the walking frame while walking.
On uneven ground	Due to their poorer sense of balance, elderly people are more likely to fall. Moreover, the walking frame would swing and become difficult to control.
Going uphill or downhill, climbing the stairs	Due to their weaker and less well-coordinated leg muscles, elderly individuals were clumsier when moving uphill and downhill or going up and down stairs. Since it is difficult to carry a walking frame forward, old people need others to bring the frame to the destination. They walk slowly forward while holding onto a handrail. If there is no handrail, they would need someone to support them. After walking, they have to sit down and rest.
Obstacles	They often stopped and took a short break before walking again.
Carrying heavy objects	Due to the weakened functioning of their body, especially in terms of the muscles, bones, and nervous system, elderly people could not carry heavy objects.
Getting up after sitting down for a long time	With atrophied leg muscles, especially calf muscles, and osteoporosis, it might be difficult to change from a sitting to a standing position. An elderly person can easily lose their balance. When an elderly person rises after sitting or squatting for a long time, they may feel dizzy.
Long duration of walking	Old people often move in a stop-and-start manner.

## Data Availability

All data are available upon request to the corresponding author, Chunjing Tao, via e-mail (chunjingtao@buaa.edu.cn).
